# Laparoscopic gastrectomy for stage II and III advanced gastric cancer: long‑term follow‑up data from a Western multicenter retrospective study

**DOI:** 10.1007/s00464-021-08505-y

**Published:** 2021-04-20

**Authors:** Umberto Bracale, Giovanni Merola, Giusto Pignata, Jacopo Andreuccetti, Pasquale Dolce, Luigi Boni, Elisa Cassinotti, Stefano Olmi, Matteo Uccelli, Monica Gualtierotti, Giovanni Ferrari, Paolo De Martini, Miloš Bjelović, Dragan Gunjić, Vania Silvestri, Emanuele Pontecorvi, Roberto Peltrini, Felice Pirozzi, Diego Cuccurullo, Antonio Sciuto, Francesco Corcione

**Affiliations:** 1 Department of General Surgery and Specialty, School of Medicine University, Federico II of Naples, Naples, Italy; 2 Department of General Surgery II, Spedali Civili of Brescia, Brescia, Italy; 3 Department of General and Mininvasive Surgery, San Camillo Hospital of Trento, Trento, Italy; 4 Department of Public Health, Federico II University of Naples, Naples, Italy; 5 Department of Surgery, Fondazione IRCCS Ca’ Granda, Ospedale Maggiore Policlinico, University of Milan, Milan, Italy; 6 Department of General and Oncologic Surgery, Centre of Advanced Laparoscopic Surgery, Centre of Bariatric Surgery, San Marco Hospital GSD, Zingonia, BG Italy; 7 Department of Minimally Invasive Oncologic Surgery, Niguarda Hospital, ASST Grande Ospedale Metropolitano Niguarda, Milan, Italy; 8 Department for Minimally Invasive Upper Digestive Surgery, Hospital for Digestive Surgery, Clinical Center of Serbia, School of Medicine, University of Belgrade, Belgrade, Serbia; 9 Department of General Surgery, Santa Maria Delle Grazie Hospital, Pozzuoli, NA Italy; 10 Department of General, Mini-Invasive and Robotic Surgery, Monaldi Hospital, Naples, NA Italy

**Keywords:** Laparoscopy, Laparoscopic gastrectomy, Advanced gastric cancer, Malignancy, Stomach

## Abstract

**Introduction:**

There has been an increasing interest for the laparoscopic treatment of early gastric cancer, especially among Eastern surgeons. However, the oncological effectiveness of Laparoscopic Gastrectomy (LG) for Advanced Gastric Cancer (AGC) remains a subject of debate, especially in Western countries where limited reports have been published. The aim of this paper is to retrospectively analyze short- and long-term results of LG for AGC in a real-life Western practice.

**Materials and methods:**

All consecutive cases of LG with D2 lymphadenectomy for AGC performed from January 2005 to December 2019 at seven different surgical departments were analyzed retrospectively. The primary outcome was diseases-free survival (DFS). Secondary outcomes were overall survival (OS), number of retrieved lymph nodes, postoperative morbidity and conversion rate.

**Results:**

A total of 366 patients with stage II and III AGC underwent either total or subtotal LG. The mean number of harvested lymph nodes was 25 ± 14. The mean hospital stay was 13 ± 10 days and overall postoperative morbidity rate 27.32%, with severe complications (grade ≥ III) accounting for 9.29%. The median follow-up was 36 ± 16 months during which 90 deaths occurred, all due to disease progression. The DFS and OS probability was equal to 0.85 (95% CI 0.81–0.89) and 0.94 (95% CI 0.92–0.97) at 1 year, 0.62 (95% CI 0.55–0.69) and 0.63 (95% CI 0.56–0.71) at 5 years, respectively.

**Conclusion:**

Our study has led us to conclude that LG for AGC is feasible and safe in the general practice of Western institutions when performed by trained surgeons.

Gastric cancer is the fifth most common malignancy and the third cause of cancer death worldwide [1]. There is a wide difference in terms of geographical prevalence, with most cases occurring in Japan, Korea and China [2]. Almost one million new cases were diagnosed globally in 2012, with more than 700,000 deaths. Of these, about 140,000 cases and more than 100,000 deaths occurred in Europe [3].

Standard gastrectomy is the main surgical procedure with curative intent for gastric cancer [4]. Since the first report of laparoscopic gastrectomy (LG) for cancer in 1994 [5], there has been an increasing interest, especially among the Eastern surgeons, for minimally invasive approach because of short-term advantages over open surgery [6]. These benefits as well as the oncological safety of LG have been clearly demonstrated for early gastric cancer. On the other hand, despite the adequate short-term outcomes, the oncological effectiveness of laparoscopic surgery for advanced gastric cancer (AGC) remains a subject of debate [7]. Indeed, several studies demonstrated the safety, feasibility and oncological value of LG compared with open gastrectomy for AGC, but most of the data comes from Eastern high-volume referral centers [8–11]. On the contrary, limited reports from the Western institutions have been published since the first and only randomized study by Huscher et al. [12] back in 2005.

The aim of this paper is to analyze short- and long-term results of LG for AGC in a real-life Western practice.

## Materials and methods

The study was approved by independent ethics committee or institutional review board of each participating institution. All candidates involved in this study provided written informed consent to manage their data. Using a prospective database, all consecutive cases of laparoscopic either total (LTG) or subtotal (LSG) gastrectomy with D2 lymphadenectomy for AGC performed from January 2005 to December 2019 at seven different departments of surgery were analyzed retrospectively.

All procedures were performed by fully trained surgeons with extensive experience in surgical oncology as well as laparoscopy.

In order to obtain a quality control of the surgical procedures, non-edited videos of both LTG and LSG performed by each participant were reviewed by the study coordinator before inclusion. Tumor stages were updated according to the 8th edition of American Joint Committee on Cancer (AJCC) TNM staging system for gastric cancer [13], and only stage II and III AGC cases were analyzed.

Preoperative (age, sex, BMI, previous abdominal surgery, ASA score, neoadjuvant therapy), intraoperative (operative time, complications, conversion rate, lymphadenectomy, type of anastomosis), and postoperative short-term (return of bowel function, hospital stay, complications, reoperation and mortality rates, TNM stage [4], number of harvested and metastatic lymph nodes) and long-term (overall survival (OS), disease-free survival (DFS), recurrence rate) data were reviewed.

Thirty-day postoperative complications were graded according to the Clavien–Dindo classification [14]. Anastomotic leakage was evaluated in accordance with the definition and grading system of the UK Surgical Infection Study Group [15].

Preoperative staging included upper gastrointestinal endoscopy and abdominal and thoracic computed tomography (CT) while endoscopic ultrasonography was not routinely performed. Since 2011 neoadjuvant chemotherapy was performed in case of cN+ or ≥ cT2 tumor. All procedures started with a Diagnostic Laparoscopy (DL) in order to detect a possible carcinomatosis. Adjuvant chemotherapy was carried out for patients with stage ≥ IB [3]. Every patient was followed-up every 6 months during the first 2 years and annually thereafter according to the local protocols.

Locoregional recurrence was defined as recurred carcinoma of the remnant gastric pouch or at anastomosis site or within the lymphatic drainage area of the region of the primary tumor, confirmed by CT scan and/or pathological examination. Distant metastases were defined as recurrent tumors in the peritoneum, liver, non-regional lymph nodes, or outside the abdominal cavity such as lung, bones, etc.

The primary outcome was DFS. Secondary outcomes were OS, number of retrieved lymph nodes, postoperative morbidity and conversion rate.

### Neoadjuvant chemotherapy

Neoadjuvant chemotherapy regimens consisted of a combination of Epirubicin—Cisplatin—5-fluorouracil—Folinic Acid (ECF, 50 mg/m^2^ epirubicin, 60 mg/m^2^ cisplatin, and 5-FU administered either by continuous infusion 200 mg/m^2^/day per 7 days via a CVC, administered every 3 weeks) or 5-fluorouracil—Folinic Acid—Oxaliplatin—Docetaxel (FLOT, docetaxel (60 mg/m^2^), oxaliplatin (85 mg/m^2^), leucovorin (200 mg/m^2^), and 5-fluorouracil (2600 mg/m^2^ as a 24 h infusion), all given on day 1 and administered every 2 weeks).

### Statistical analysis

Data are presented as median (I quartile-Q1; III quartile-Q3) or mean ± standard deviation (SD) for quantitative variables as appropriate and as number of patients (%) for qualitative variables.

Actuarial OS was calculated as the time from surgery to death using Kaplan–Meier methods. Differences between curves were tested using the Log-Rank test. Univariate and multivariate Cox proportional hazard regression analysis was performed to estimate the hazard ratios for OR.

All statistical analyses were performed using R 3.6.0 software. Survival analysis was performed using survival package, version 2.44-1.1. Statistical significance was predetermined as *p* < 0.05. All tests were two-tailed and statistical significance was set at an α level of 0.05.

### Surgical technique

#### Laparoscopic subtotal gastrectomy (LSG)

Four trocars were placed in the upper abdomen. Following abdominal exploration and omentectomy, lymph node dissection was performed including D1 (stations 1–7) + 8a (common hepatic artery), 9 (celiac), 11p (proximal splenic artery) and 12a (anterior hepato-duodenal ligament) stations [16]. The stomach was sectioned using a linear stapler with the aim to achieve a proximal margin of 5 cm. Either Roux-en-Y or Billroth II anastomosis was performed. A side-to-side gastro-jejunal anastomosis was performed with a linear stapler.

#### Laparoscopic total gastrectomy (LTG)

The technique was the same for LSG until section of the stomach [17, 18]. Lymph node dissection included also the 11d (distal splenic artery) station. The gastroesophageal junction was sectioned using a linear stapler and then a Roux-en-Y reconstruction was always performed. Oesophago-jejunal anastomosis was performed using either a circular or a linear stapler (like Orringer) [19, 20].

## Results

### Patients demographics, types of gastrectomy and pathologic features

From January 2005 to December 2019, a total of 654 patients underwent either LTG or LSG at seven different surgical units. Three hundred and sixty-six patients were analyzed according to the inclusion criteria. Patient characteristics and pathologic features are reported in Table 1.

**Table 1:** Biometric and pathological features, intra- and postoperative data

	Patients (*n* = 366)	Laparoscopic total gastrectomy (*n* = 109)	Laparoscopic subtotal gastrectomy (*n* = 257)
Sex (M/F)	204/162	73/36	131/126
Age (mean ± SD) years	68.4 ± 11.6	66.1 ± 11.3	69.4 ± 11.7
BMI (mean ± SD) Kg/m^2^	24.8 ± 4.1	24.5 ± 3.6	24.9 ± 4.3
Previous abdominal surgery			
Cholecystectomy	18 (5%)	6	12
Appendectomy	24 (6.6%)	13	11
Hysterectomy	9 (2.5%)	5	4
Left colectomy	4 (1.1%)	1	3
Right colectomy	2 (1%)	1	1
ASA			
I	42 (11.5%)	17	25
II	212 (57.9%)	64	148
III	111 (30.3%)	27	84
IV	1	1	0
Stage^a^			
IIa	80 (21.8%)	25	55
IIb	64 (17.5%)	19	45
IIIa	126 (34.4%)	45	81
IIIb	77 (21%)	48	29
IIIc	19 (5.1%)	7	12
Neoadjuvant therapy	97 (26%)	30	67
OrVil/Orringer anastomosis		3/106	
Roux-en-Y/Billroth II	342/24	109/0	233/24
Operative time (mean ± SD) min	247.7 ± 105.3	273.8 ± 114.9	236.6 ± 99.1
Nodes harvested (median ± IQR)	25 ± 14	25 ± 10	27 ± 11
Metastatic nodes (median ± IQR)	1 ± 5	3 ± 4	2 ± 3
Intraoperative complications *n* (%)	21 (5.7%)	11 (3%)	10 (2.7%)
Conversion *n* (%)	53 (14.5%)	32 (8.7%)	21 (5.7%)
Lymphadenectomy *n* (%)			
D2	352 (96.17%)	103	249
D2+	14 (3.82%)	6	8
Overall postoperative complications *n* (%)	100 (27.3%)	32 (8.7%)	68 (18.57%)
Postoperative complications			
II	66	18	48
IIIa	8	3	5
IIIb	20	10	10
IV	3	1	2
V	3	0	3
Bowel recovery (mean ± SD) days	3.6 ± 2.3	3.9 ± 2.3	2.4 ± 2.3
Length of stay (median ± IQR) days	13 ± 10	15 ± 12	12 ± 8
Death related to disease progression *n* (%)	90 (24.6%)	35 (9.58%)	55 (15.02%)
Follow-up (median ± IQR; range) months	36 ± 16 (range 1–80 months)		

*IQR* interquartile range*, BMI* body mass index, *ASA* American Society of Anesthesiologists

^a^AJCC TNM staging, 8th edition

LSG and LTG were performed in 257 (70.8%) and 109 (29.2%) cases, respectively. D2 dissection was performed in 352 (96.2%) cases, while D2 + dissection was performed in 14 (3.8%) cases.

The mean operative time was 247.7 ± 105.3 min and conversion rate 14.5% (53/366). Conversions were due to extensive adhesions (25 cases), accidental spleen injury (6 cases), bleeding (10 cases) and bulky tumors or cancers invading serosa or adjacent structures (12 cases). Intraoperative data are reported in Table 1.

The median number of harvested lymph nodes was 25 ± 14. Cancer pathologic stages were: IIA in 80 (21.8%) patients, IIB in 64 (17.5%) patients, IIIA in 126 (34.4%) patients, IIIB in 77 (21%) patients and IIIC in 19 (5.1%) patients.

### Postoperative complications and treatment

The mean length of hospital stay was 13 ± 10 days and overall postoperative morbidity rate was 27.32% (100/366) with severe complications (grade ≥ III) accounting for 9.29% (Table 1).

Grade IIIa complications included an esophago-jejunal leak and 7 cases of anastomotic bleeding, which were all managed endoscopically, while operative management was needed in 20 cases (grade IIIb) including anastomotic leakage (9 cases), duodenal stump leakage (4 cases), abdominal bleeding (5 cases), internal hernia (1 case) and transverse colon ischemia (1 case). We reported 3 grade V complications, accounting for a postoperative mortality of 0.81%. Details about postoperative complications and their management are reported in Tables 1 and 2. Logistic binary regression for complications adjusted for other effects (Table 3) showed at univariate analysis a statistically significant difference in patients who underwent neoadjuvant therapy (OR 2.15; *p* value 0.03). This difference was confirmed at multivariate analysis (OR 2.17; *p* value 0.04).

**Table 2:** Postoperative complications and their treatment after laparoscopic gastrectomy

Postoperative complication	*n* (%)	Treatment
Total	100 (27.32%)	
Ileuss	13 (3.55%)	13 nasogastric tube re-positioning
Urinary infection	15 (4.09%)	15 antibiotic therapy
Wound infection	16 (4.37%)	16 antibiotic therapy
Bleeding	15 (4.09%)	5 laparoscopic hemostasis 10 blood transfusion
Lung morbidity	15 (4.09%)	3 pulmonary embolisms needed intensive care unit therapy 12 pulmonary infections needed antibiotic therapy
Esophago-jejunal bleeding	4 (1.09%)	All solved with endoscopic hemostasis
Gastro-jejunal bleeding	3 (0.81%)	All solved with endoscopic hemostasis
Esophago-jejunal leakage	8 (2.18%)	4 laparoscopic anastomotic re-do 1 endoscopic stent placement 3 open anastomotic re-do
Gastro-jejunal leakage	1 (0.27%)	1 laparoscopic anastomotic re-do
Jejuno-jejunal-leakage	1 (0.27%)	1 laparoscopic anastomotic re-do
Duodenal leakage	4 (1.09%)	2 laparoscopic peritoneal toilettes, suture of duodenum and drainage 2 open peritoneal toilettes, suture of duodenum and drainage
Internal hernia	1 (0.27%)	1 laparoscopic internal hernia reduction and mesentery defect closure
Transverse colon ischemia	1 (0.27%)	1 open transverse colon resection
Postoperative mortality	3 (0.82%)	All due to heart failure

**Table 3:** Logistic binary univariate and multivariate regression with postoperative complication as dependent item

	Postoperative complications		Univariate analysis		Multivariate analysis	
	Yes *n* = 100 (%)	No *n* = 266 (%)	OR	*p* value	aOR	*p* value
Sex						
F	37 (37%)	125 (47%)	0.66	0.087	0.621	0.064
M	63 (63%)	141 (53%)	/	/	/	/
Age	69.6 ± 12.9	67.9 ± 11.1	1	0.209	1	0.238
BMI	24.3 ± 4.42	24.96 ± 4	0.96	0.238	1	0.192
ASA						
3	33 (33%)	79 (29.7%)	1.13	0.5	1.05	0.8
2	57 (57%)	155 (58.3%)	/	/	/	/
1	10 (10%)	32 (12%)	/	/	/	/
Operative time	257.7 ± 105.2	243.9 ± 105.2	1	0.266	1	0.26
Procedure						
LTG	33 (33%)	76 (28.6%)	0.812	0.4	1.03	0.9
LSG	67 (67%)	190 (71.4%)	/	/	/	/
Conversion						
Yes					1	0.9
No			/	/	/	/
Blood loss	104 ± 91	91 ± 68			1	0.116
Intraop. complication						
Yes	7 (7%)	14 (5.3%)	0.73	0.52	0.5	0.62
No	93 (93%)	252 (94.7%)	/	/	/	/
Harvested nodes	25 ± 9	27 ± 11	1	0.13	1	0.214
Metastatic nodes	3 ± 4	3 ± 4	1	0.821	1	0.773
Stage						
III	60 (60%)	162 (60.9%)	1.03	0.87	0.7	0.25
II	40 (40%)	104 (39.1%)	/	/	/	/
Neoadjuvant therapy						
Yes	38 (38%)	59 (22.2%)	2.15	0.03	2.17	0.04
No	62 (62%)	207 (77.8%)	/	/	/	/

Data are reported as number of patients (%) or mean (± standard deviation) or median (± IQR). OR and corresponding *p* values are obtained using logistic binary univariate and multivariate regression

### Survival and recurrence

**Fig. 1:**
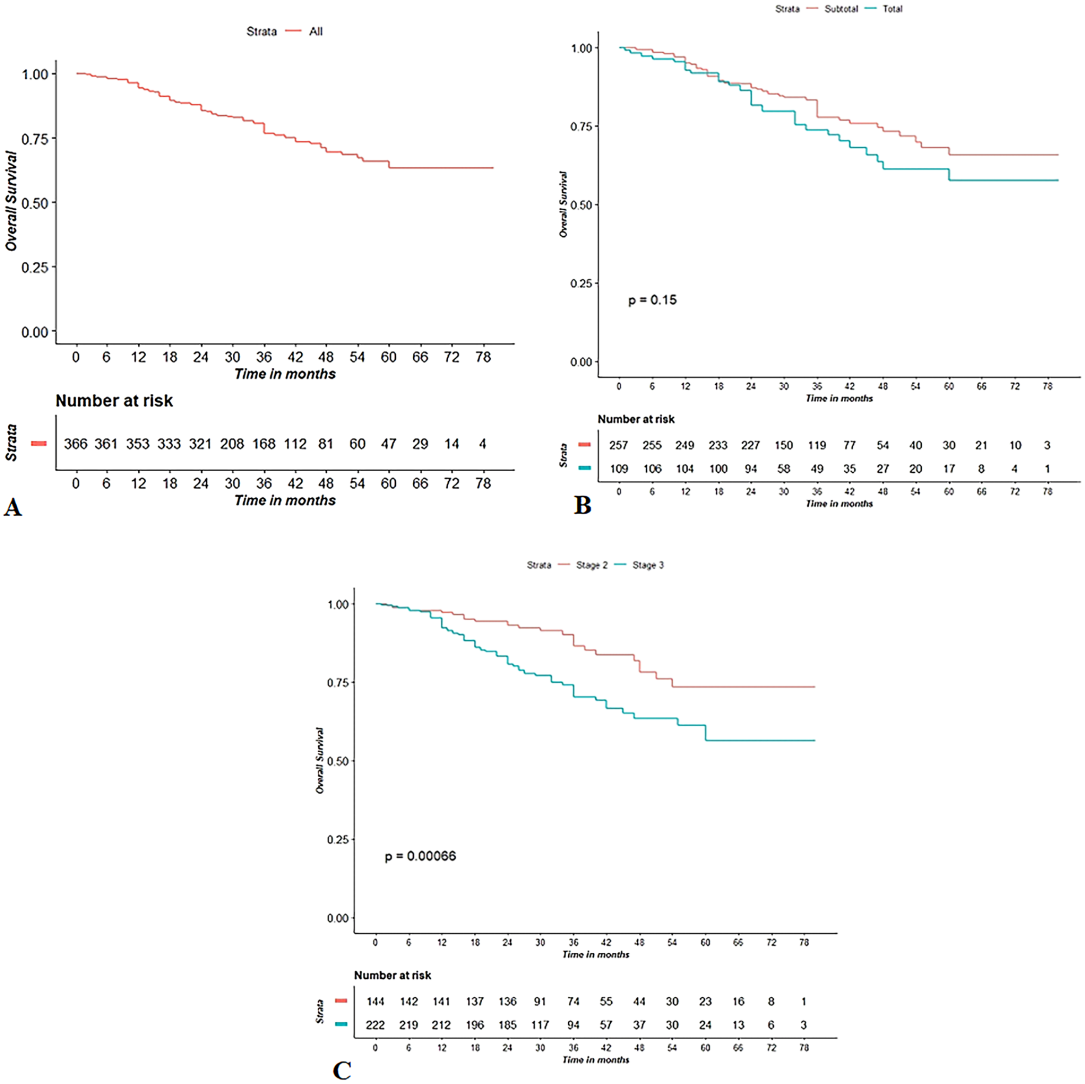
Kaplan–Meier curve for OS (**A**); Kaplan–Meier curve for OS, stratifying for procedure (**B**) and stage (**C**)

The median follow-up was 36 ± 16 months (range 1–80 months) during which 90 deaths occurred, all due to disease progression. Kaplan–Meier OS curve is presented in Fig. 1a. The OS probability was equal to 0.94 (95% CI 0.92–0.97) at 1 year and 0.63 (95% CI 0.56–0.71) at 5 years. Kaplan–Meier OS curves (Fig. 1b, c) were also reported stratifying for procedure (LSG vs. LTG) and stage (II vs. III). The OS probability for stage II was 0.97 (95% CI 0.94–0.99) at 1 year and 0.73 (95% CI 0.63–0.85) at 5 years. The OS probability for stage III was 0.92 (95% CI 0.89–0.96) at 1 year and 0.56 (95% CI 0.46–0.68) at 5 years. The corresponding log-rank tests for LSG vs. LTG and stage II vs. stage III demonstrated a non-significant (*p* = 0.15) and a significant difference (*p* = 0.0006), respectively. However, at multivariate Cox regressions, both differences were found statistically non-significant. Table 5 shows the results for univariate and multivariate Cox regression analysis for OS. Except for metastatic nodes (aHR = 1.06, *p* < 0.009), all the other variables were not significantly associated to OS at multivariate analysis (Table 4).

**Table 4:** Cox regression model analysis for overall survival (OS)

	Death		Univariate analysis		Multivariate analysis	
	Yes (*n* = 90)	No (*n* = 276)	HR	*p* value	a-HR	*p* value
Procedure						
LTG	35 (38.9%)	74 (26.8%)	1.36	0.15	1.24	0.34
LSG	55 (61.1%)	202 (73.2%)	/	/	/	/
Conversion						
Yes	20 (22.2%)	33 (12%)	1.37	0.349	1.27	0.38
No	70 (77.8%)	243 (88%)	/	/	/	/
Harvested nodes	26.2 ± 11.0	26.9 ± 10.77	1	0.75	1	0.74
Metastatic nodes	4.99 ± 5.13	2.53 ± 3.84	1.1	< 0.001	1.06	0.009
Stage						
III	66 (73.3%)	156 (56.5%)	2.2	0.001	1.5	0.15
II	24 (26.7%)	120 (43.5)	/	/	/	/
Neoadjuvant therapy						
Yes	29 (32.2%)	68 (24.6%)	1.4	0.09	1.2	0.44
No	61 (67.8%)	208 (75.4)	/	/	/	/

Data are reported as number of patients (%) or mean (± standard deviation). HR and corresponding *p* values are obtained using Cox regression analysis

The overall recurrence rate was 28.6% (105/366). There were 32/366 (8.7%) cases of locoregional recurrence, including 15 (4%) of regional lymph node involvement, 10 (2.7%) in the remnant stomach and 7 (2%) at the anastomotic site. Distant metastases were found in 60/366 (16.4%) cases at the following sites: peritoneum (26 cases, 7.1%), liver (21 cases, 5.7%), other organs (8 cases, 2.1%), non-regional lymph nodes (5 cases, 1.4%). Recurrence at both locoregional and distant sites occurred in 13/366 (3.5%) cases.

**Fig. 2:**
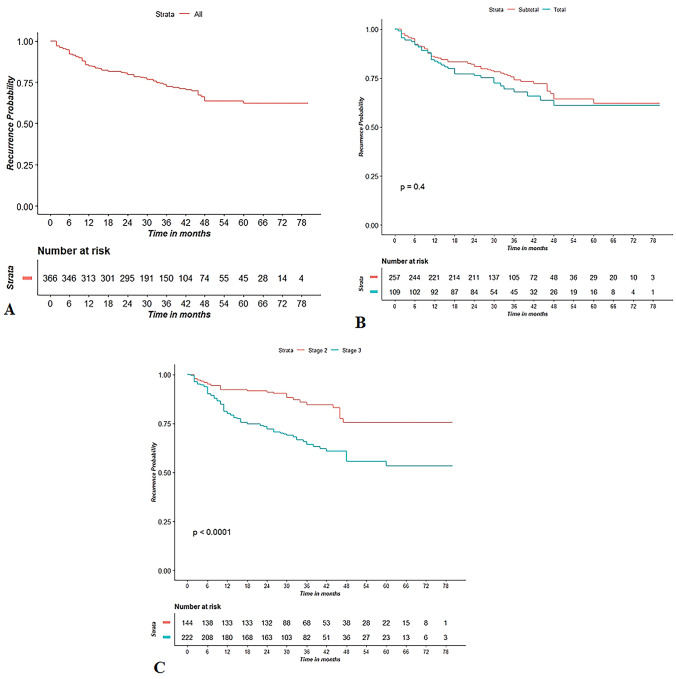
Kaplan–Meier curve for DFS (**A**); Kaplan–Meier curve for DSF, stratifying for procedure (**B**) and stage (**C**)

DFS probability was 0.85 (95% CI 0.81–0.89) at 1 year and 0.62 (95% CI 0.55–0.69) at 5 years (Fig. 2a). Kaplan–Meier DFS curves (Fig. 2b, c) were also reported stratifying for procedure (LSG vs. LTG) and stage (II vs. III) (Table 5).

**Table 5:** Cox regression model analysis for disease-free survival (DFS)

	Recurrence		Univariate analysis		Multivariate analysis	
	Yes *n* = 105 (%)	No *n* = 261 (%)	HR	*p* value	a-HR	*p* value
Procedure						
LTG	35 (33.3%)	74 (28.3%)	1.2	0.4	1	0.75
LSG	70 (67.7%)	187 (71.7%)	/	/	/	/
Conversion						
Yes	24 (22.8%)	29 (11.11%)	1.9	0.003	1.3	0.26
No	81 (77.2%)	232 (88.9%)	/	/	/	/
Harvested nodes	26 ± 11	27 ± 11	1	0.76	1	0.74
Metastatic nodes	5 ± 5	2 ± 4	1.1	< 0.001	1.06	0.003
Stage						
III	81 (77.2%)	141 (54%)	2.5	< 0.001	1.75	0.037
II	24 (22.8%)	120 (46%)	/	/	/	/
Neoadjuvant therapy						
Yes	33 (31.4%)	64 (24.5%)	1.3	0.2	1	0.8
No	72 (68.6%)	197 (75.5%)	/	/	/	/

Data are reported as number of patients (%) or mean (± standard deviation). HR and corresponding *p* values are obtained using Cox regression analysis

## Discussion

Radical gastrectomy still represents the treatment of choice for gastric cancer. In case of early gastric cancer, considering the reduced risk of node metastases and local recurrence [1], LG is deemed the gold standard approach, especially in most of Eastern countries [4, 21]. This recommendation comes from the results of some randomized trials which demonstrated a lower morbidity and a non-inferior OS than the open approach [22, 23].

More recently, some Eastern studies seem to confirm that LG can be also considered for the treatment of AGC [24–26]. The Chinese CLASS-01 trial investigated the short-term surgical outcomes of 1056 patients with T2-4aN0-3M0 cancer at 14 centers, reporting similar postoperative morbidity and mortality as well as severity of complications for laparoscopic and open D2 distal gastrectomy [26]. Long-term results from this trial were reported in 2019, demonstrating as non-inferior the 3-year DFS of patients assigned to the laparoscopic group than those assigned to the open group [26]. Other ongoing trials as JLSSG0901 [27] and KLASS-02 [28] reported similar or lower complication rates, less postoperative pain and faster recovery for laparoscopic vs. open distal gastrectomy, while the long-term oncologic outcomes are still awaited.

It is unclear whether these findings can be applied to Western patients, who tend to have more advanced disease at presentation and a higher incidence of proximally located as well as diffuse-type cancers than Eastern patients. Furthermore, surgeon and hospital volumes of laparoscopic gastrectomy are significantly higher in the East than in the West [12]. Thus, high-quality evidence from large-scale studies on LG for AGC is lacking in Western countries, and most series include both early and advanced cancers, thus hampering a stage-based analysis [29–36].

In 2005 Huscher et al. published the only European randomized trial comparing laparoscopic and open subtotal gastrectomy for distal gastric cancer. In their analysis on 59 patients, LG was found to be a feasible and safe oncologic procedure with short- and long-term results similar or better than those of open surgery [12]. The results from the LOGICA trial [37] and the STOMACH trial [38] are still expected.

Finally, staging, surgical training and use of adjuvant therapy are different in non-tertiary referral centers [26].

For all the above reasons, well-controlled clinical studies may not reflect the actual outcome of laparoscopy for AGC in the clinical practice [8], and results from our study do provide real-life data on the safety and oncologic efficacy of LG in a large series of patients from different Western institutions.

We recorded an overall postoperative morbidity of 27.3%, but the majority (66%) of complications were classified as grade II requiring only a pharmacological treatment. These results are consistent with those of most published Western series. Huscher et al. reported a morbidity rate of 26.7% following laparoscopic distal gastrectomy [12]. Also, several other retrospective studies reported an incidence of postoperative complications ranging from 25 to about 32% [29–36]. It has been suggested that the laparoscopic approach may decrease the incidence of minor complications in the early and late postoperative periods compared with the open approach [32]. In a recent study from Korea on 1483 laparoscopic gastrectomies for AGC, the overall morbidity rate was 9.1% with 54% of complications being classified as grade ≥ 3. At the multivariate analysis, age was found to be associated with postoperative morbidity, endorsing that extended surgery, although minimally invasive, may be risky for the elderly [8]. Other predictive factors for complications have been suggested, including sex, comorbidity, type of resection and surgeon’s experience [26]. Our study experience suggests that neoadjuvant therapy may lead to an increased risk of postoperative complications. Although this observation has not been confirmed by several other investigators [39–41], the analysis of the CRITICS gastric cancer trial revealed a morbidity rate as high as 47% in patients receiving neoadjuvant chemotherapy [42]. On the other hand, it has been suggested that preoperative chemotherapy may abolish the poor prognosis induced by postoperative complications after curative resection [43].

We reported a 14.5% conversion rate. However, 40 conversions were recorded during the first ten years of the study period while, over the last five years, the rate decreased to 3.6% which favorably compares to that of some previous reports, ranging from 2.2 to 7% [11, 44–46]. Also, a conversion rate up to 17.4% has been reported following LTG in large series [47]. Our finding is probably related to the improved technical skills and experience of the surgeons over the study period as well as to prompt consideration of conversion when concerned for adequate oncologic resection [32]. The most common reason for conversion was a technical factor such as adhesions, bleeding during difficult lymphadenectomy, whereas tumor factors (bulky/T4) accounted for about 23% of conversions.

All procedures started with a DL in order to detect a possible carcinomatosis. DL represents the first step during LG. It plays an important role in avoiding unnecessary laparotomies, particularly in cases of AGC. Many studies showed that DL demonstrated moderate to substantial agreement with final pathology for T stage, but only fair agreement for N stage. For M staging, DL had an overall accuracy, sensitivity, and specificity ranging from 85–98.9%, 64.3–94%, and 80–100%, respectively [18].

D2 lymph node dissection is of paramount importance for curative gastrectomy, but due to the technical difficulties it has limited enthusiasm for laparoscopic approach to AGC [33]. Concern about achieving adequate lymph node retrieval has been raised in some earlier series, where up to 38% of patients had less than the AJCC minimum number of lymph node harvest needed for proper staging [48]. In our experience, the mean number of 25 harvested nodes allowed us to meet the criteria for adequate laparoscopic lymphadenectomy in AGC [12]. Since early 2000s, there have been many controversies on the performance of splenectomy, and the prognostic value of lymphadenectomy of the n.10 station is debated [1]. In accordance with the SIC-GIRCG 2013 Consensus Conference on Gastric Cancer, radical excision of the splenic hilum lymph nodes or splenectomy was reserved for AGC cases of the upper greater curvature, in which the malignancy was suspected to be T4 or there were suspected nodes at splenic hilum [16]. Similarly, according to the most recent Japanese guidelines, dissection of n. 10 nodes/splenectomy is excluded from standard D2 dissection, unless a tumor of the upper stomach invades the greater curvature or there are metastases to no. 4sb lymph nodes [4]. In those cases, we believe that the open approach should be preferred [12].

In our series, the OS probability was 0.94 at 1 year and 0.63 at 5 years, while the DFS probability was 0.85 at 1 year and 0.62 at 5 years. These figures favorably compare to those of the few Western studies which analyzed survival data. In their series of 30 laparoscopic cases with 57% of stage ≥ II cancers, Huscher et al. found 5-year OS and DFS rates of 58.9% and 57.3%, respectively [12]. Similar results have been reported in a series of 70 AGC (stage IB-IV) patients undergoing laparoscopic gastrectomy [12]. Kelly et al. reported higher rates of 81% and 85% in their cohort of 87 patients; however, only 37% of tumors were stage II and III [12]. Three-year survival data have been analyzed in a series of 21 AGC patients, with an OS of 69.5% and a relapse-free survival of 44.5% [48]. At 5 years, we found an OS probability of 0.73 for stage II and 0.56 for stage III. Survival data for cancer stage are mainly available Eastern studies. In their retrospective analysis of a 15-year experience, Min et al. reported a 5-year OS of 88.7% for stage IIA, 84.2 for stage IIB and 60.3% for stage III, with a significant difference between stages IIIA, IIIB and IIIC [8].

We reported an overall recurrence rate of 28.6%, which is in the range of 13.3% to 50% reported by other authors [29–36, 48, 49]. Peritoneal involvement has been reported as the most common type of recurrence after LG in several Eastern series [50], while recurrence patterns in Western patients have not been well established [48]. In our study, the most common sites of recurrence were peritoneum (7.1%), liver (5.7%) and regional lymph nodes (4%). Similarly, in a series of 21 patients, the recurrence rate was 38.1% (8/21); peritoneal recurrence was recorded in 19% (4/21), distant recurrence in 14.3% (3/21) and a mixed pattern (both locoregional and distant) in 4.8% (1/21) [48]. Strong et al. [36] found an equal distribution of local (*n* = 2, 6.6%) and distant (*n* = 2, 6.6%) recurrence in a cohort of 30 patients. Sica et al. [49] reported 11 cases (11/22, 50%) of recurrence after a median follow-up of 39 months, with hepatic metastases being the most common (6/22, 27.3%), followed by other distant recurrences (3/22, 13.7%) and locoregional recurrence (2/22, 9%). Some authors argue that while distant metastases after LG can be explained by invisible micro-metastasis during or before surgery, local recurrence may be associated with the adequacy of surgery [8].

Many doubts still concern the role of neoadjuvant chemotherapy for gastric cancer [50–52].

In the present study, neoadjuvant chemotherapy is not associated with recurrence or death. This finding is consistent with the EORTC trial [53] that failed to demonstrate prognostic benefits regarding OS after neoadjuvant chemotherapy, despite a significantly increased R0 resection rate. Furthermore, only 26% of patients in our series received neoadjuvant treatment. Finally we argue that a quality of surgery with adeguate lymphadenectomy performed by experienced surgeons could minimize the risk of metastatic nodes.

Our study has some limitations mainly related to the retrospective design. Data have been collected from different institutions over a 15-year period. Thus, treatment protocols and perioperative management were not standardized. No patients received neoadjuvant therapy until 2010, which can influence postoperative outcomes. Moreover, there has been much development of procedures and technologies over time, and this does influence the data. Also, although proficiency of participating surgeons has been established, their experience and technical skill increased as time passed. However, these limitations are inherent in that the study provides a true representation of outcomes in a general practice setting.

In conclusion, our study has led us to conclude that laparoscopic gastrectomy for advanced gastric cancer is feasible and safe in the general practice of Western institutions when performed by trained surgeons. Similarly, some caution must be exercised when translating the current evidence also on Robotic Assisted Gastrectomy (RAG) to a European population. Benefits of RAG include the use of ICG to assess vascularity and (sentinel) lymph nodes. Inclusion of artificial intelligence and machine learning to aid the surgeon in these complex procedures are coming on the horizon [54]_._

Randomized controlled trials carried out in this setting are needed to corroborate our results.
